# Community Perception and Attitude towards people with Depression among Adults Residing in Arba Minch Health and Demographic Surveillance Site (AM-HDSS), Southern Ethiopia

**DOI:** 10.4314/ejhs.v30i4.12

**Published:** 2020-07-01

**Authors:** Negussie Boti1, Sultan Hussen, Gistane Ayele, Abera Mersha, Selamawit Gebeyehu, Mekidm Kassa, Tesfaye Feleke, Bilcha Oumer

**Affiliations:** 1 Arba Minch University, College of Medicine and Health Sciences, School of Public Health, Arba Minch, Southern Ethiopia; 2 Arba Minch University, College of Medicine and Health Sciences, School of Nursing, Arba Minch, Southern Ethiopia; 3 Arba Minch Health Sciences College, Department of Midwifery, Arba Minch, Southern Ethiopia

**Keywords:** Perception, Attitude, Depression, AM-HDSS, Ethiopia

## Abstract

**Background:**

Negative perception and attitude of community prevents many people with depression and their caregivers from seeking help and receiving adequate treatment due to fear of social reaction and may try to hide the illness. The reasons for negative attitudes are not consistent across communities or cultures. Therefore, understanding the level of community perception and attitude towards people with depression is important to develop an intervention to reduce the impact of mental illness.

**Methods:**

A community-based cross-sectional study was conducted among 617 randomly selected adults. The data was collected using structured, pre-tested, and interviewer-administered questionnaires. Descriptive statistics like frequency, mean, and median were performed. Bi-variable and multivariable logistic regression analyses were performed to identify the factors that affect the community attitude towards people with depression.

**Results:**

Of the study population, 325(52.7%) had a good perception and 246(39.9%) had a positive attitude towards people with depression. The majority of study participants frequently identified as the perceived cause of depression was substance misuse, loss of loved one, and conflict within a family. In addition, psychosocial treatment was the most preferred treatment for people with depression in the study area. Marital status and educational status were significantly associated with the community attitude towards people with depression

**Conclusion:**

Giving special attention to people with substance misuse, loss of loved one, and conflict within a family is very vital for the prevention of depression. In addition, future mental health promotion activities should focus on cause and common manifestation of depression to improve the attitude toward people with depression.

## Introduction

According to the World Health Organization's (WHO) definition, “Mental health is a state of well-being in which an individual can realize his or her abilities, interact positively with others, cope with the stressors of life and study, work productively and fruitfully, and contribute to his or her family and community.” It should be noted that the definition does not refer exclusively to the absence of “mental illness”, but also addresses the concept of “mental wellness” (1). World Health Organization (WHO) defines “Depressive disorders are characterized by sadness, loss of interest or pleasure, feelings of guilt or low self-worth, disturbed sleep or appetite, feelings of tiredness, and poor concentration” (2).

Today, depression is a global public health problem (1,2). According to WHO, globally, more than 322 million people were affected by depression (3). The burden of mental disorders continues to grow with significant impacts on health and major social, human rights and economic consequences in all countries of the world (3). Mental disorders attributed to 14% of the global burden of disease and most of the people affected (75%) are from low-income countries in which they do not have access to the treatment they need (4,5). In Africa, Ethiopia, Lesotho and Cape Verde were the three countries with a high burden of depression (3).

Even though depression was one of the public health problems in Africa, most of those patients did not get treatment due to neglect in Africa's health especially in Ethiopia (4). The “treatment gap” – the proportion of people with mental illness who do not get treatment – ranges from 75% in South Africa to more than 90% in Nigeria(4).

Evidence shows that a negative attitude is one of the barriers to seeking help for people living with depression (5, 6). In sub-Saharan Africa, stigmatizing attitudes towards mental illness leads to several detrimental consequences, especially in rural areas. So far, few studies were conducted in sub-Saharan Africa (7, 8). In Ethiopia, mental health problems have a significant effect on the economic burden of households, but most people use traditional methods for treatments of depression (9, 10). After trying traditional methods and failed, many of them keep the patient at home and few of them consult health professionals (10).

Most Ethiopians perceive that depression is an illness caused by supernatural forces (10). This influences the attitude towards mental illness and help-seeking associated with mental health problems (9, 11). In addition, several factors are associated with the community perception and attitude related to depression which is mainly socio-demographic including gender, age, religion, income and education (9, 12, 13). However, little is known about the perception and attitude of the community regarding people living with depression in Ethiopia including the study area (9, 12, 13). Therefore, assessing the current status of community perception and attitude towards people with depression may help to develop interventions that may contribute to an enabling environment to fully access and utilize mental health services. Therefore, this study aimed to assess the recent status of community perception and attitude towards people with depression among residents of Arba Minch Zuria District, AM-HDSS, and Southern Ethiopia.

## Methods

### Study area and period:

This study was conducted in the nine kebeles of Arba Minch Zuria districts included under Arba Minch Health and Demographic Surveillance sites (AM-HDSS). Arba Minch town was the zonal capital city and located at 450km from Addis Ababa and 275km from the regional town, Hawassa. The district was bordered on the south by the Dirashe special woreda, on the west by Bonke, on the north by Dita and Chencha, on the northeast by Mirab Abaya, on the east by the Oromia Region, and on the southeast by the Amaro special woreda. Arba Minch HDSS includes nine kebeles (the lowest administrative unit of Ethiopia) of Arba Minch Zuria District. Eight of the nine HDSS Kebeles are rural and the remaining one is semi-urban. According to the 2016 report from the site, 14,754 households and 74,107 individuals (males = 37,130 and females = 36,977) were living in the site.

### Eligibility criteria:

All adult residents aged (18–64 years) and lived at least 6 months in the study area during the data collection period were included in this study. Those who were critically ill during the data collection period were excluded.

### Sample size and sampling technique:

To determine the sample size for this study, we used single population proportion formula considering the following assumptions: the proportion of the community who had poor perception towards mental illness is 37.3 % (***12***), 95% confidence level (1.96), 4% margin of error, and 10% non-response rate. Therefore, the minimal sample size required for this study was 617. To select the study participants, we used a sampling frame of the AM-HDSS database. Then, based on the total number of households living in each kebele, study participants were proportionally allocated. Then, using a simple random sampling method, the study participants were selected using computer-generated random numbers.

### Measurement Community attitude toward people with depression:

It refers to a community feeling of expression towards mental illness. It was measured based on 13 attitude related questions. To categorize community attitude towards people with depression, we used the demarcation threshold formula. Those who scored ≤ 39 were considered to have good attitude towards people with depression (9, 14).

### Community perception toward people living with depression:

It refers to the level of identification, and interpretation of sensory information to represent and understand the presented information. It was assessed by 9 items of five Likert scale perception related questionnaires. To categorize community perception towards people with depression, we used the demarcation threshold formula. Those who scored ≤27 for 9 items of five semantic differential scales were considered as having a good perception towards people with depression (9, 12).

### Data collection procedure:

The data was collected by using a pre-tested structured interviewer-administered questionnaire which is developed by reviewing different literature (9, 12, 13). To assess community perception toward people with depression, we used 9 items of five Likert scale perception related questionnaires from 1 to 5 (1 = strongly agree, 2 = agree, 3 = neutral, 4 = disagree, and 5 = strongly disagree). This tool also was pretested and validated in Ethiopia and another sub-Saharan Africa (9, 12, 15). To assess community attitude towards people with depression, we used 13 items of five-point Likert scale from 1 to 5 (1 = strongly agree, 2 = agree, 3 = neutral, 4 = disagree, and 5 = strongly disagree). This tool was initially developed by the WHO expert group and previously pretested and validated in Ethiopia (9, 14).

The tools were first developed in the English language and then translated into the Amharic language then back to the English language to check the consistency. The data was collected by using ODK software. Eighty high school completed data collectors and five diploma level supervisors who had direct experience and ability to speak the Amharic language was recruited for data collection. After giving three days’ intensive training, the data was collected by going house-to-house to the randomly selected house numbers.

### Data quality control:

Data quality was ensured during instrument development, collection, coding, and analysis. Experienced data collectors and supervisors of Arba Minch HDSS were involved in the data collection process. The training was given to the supervisors and data collectors. Pretest was conducted on 5% of the sample size in Mirab Abaya, before the actual data collection. After the pretest, modifications were made on the tools based on the identified gaps. Supervisors monitored the whole data collection process and checked the data for completeness every day during the data collection time. To increase the response rate, the data collectors repeatedly visited (at least three times) those participants who were not present at the house during data collection.

### Data processing and analysis:

The collected data was cleaned, edited, and analyzed by using STATA version 14.0. A descriptive analysis was conducted. Then, binary logistic regression analysis was performed for each independent variable and outcome of interests to identify independent predictors. Upon the completion of the bivariate logistic regression analysis, variables with p-value <0.25 were selected for the multivariable analysis. Context and previous studies were also considered to make a variable candidate for multivariable analysis. To assess the strength of association, the p-value < 0.05 was used. Multi-collinearity assumption and goodness of fit test were measured using the variance inflation factor (VIF), Hosmer and Lemeshow test respectively.

### Ethical consideration:

A letter of ethical approval was obtained from the institutional review board of the College of Medicine and Health Sciences at Arba Minch University with the reference number of CMHS/12033440/111. Written consent from all participants was obtained after being fully informed about the objectives and procedures of the study for study participants. The confidentiality and privacy of participants were actively protected. All participants were assigned a unique identification number. The study participants voluntarily participated in the study. They had the right to stop or discontinue from the study at any time.

## Results

### Socio-demographic characteristics of the study participants:

A total of 617 individuals participated in this study with a response rate of 100%. Among the total 617 respondents, 324(52.5%) were males and 559(90.6%) of them were ever been married. The mean (±SD) age of respondents was 35.61 (±10.52) years. The majority of the respondents, 507(82.2%), were Gamo by ethnicity and 423(68.6%) were protestant Christians in their religion. Regarding their educational status, 312(50.6%) of the participants completed primary school, and 615(99.7%) were rural residents (Table 1).

**Table 1:** Socio-demographic characteristics of respondents residing in AM-HDSS, Southern Ethiopia, 2019 (N = 617).

Variables	Category	Frequency	Percent
Age group (Years)	18–24	170	27.60
	25–44	252	40.80
	≥45	195	31.60
Sex	Male	324	52.50
	Female	293	47.50
Religion	Orthodox	188	30.5
	Protestant	423	68.6
	Muslim	2	0.30
	Traditional	4	0.60
Ethnicity	Gamo	507	82.2
	Gofa	3	0.50
	Zayse	52	8.40
	Woliata	51	8.30
	Oromo	3	0.50
	Kore	1	0.20
Educational status	No formal education	224	36.30
	Primary (1–8)	312	50.60
	Secondary (9–12)	64	10.40
	College and above	17	2.80
Residence	Urban	2	0.30
	Rural	615	99.70
Marital status	Married	559	90.60
	Single	30	4.86
	Divorced	28	4.54
Occupational status	Farmers	311	50.40
	Merchant	52	8.40
	Governmental Employer	16	2.60
	Daily laborer	98	15.90
	Students	140	22.70
Family history of mental illness	Yes	45	7.30
	No	572	92.70
Wealth index	Low	202	32.70
	Moderate	210	34.02
	High	205	33.20

### General information about depression:

Around 597(96.8%) of the participants ever heard about depression. The main sources of information for depression were friends which accounts for 489(81.91%) followed by mass media 261(43.72%) of the respondents.

From the total study participants, 393(65.83%) listed the possible case of depression. The majority of the study participants 340(86.51%) perceived that the cause of depression is substance misuse, 240(51.91%) and 289 (46.82%) of the respondents perceived that the cause of depression is the loss of loved one and conflict within the family, respectively. Furthermore, the majority 430(72.03%) of respondents mentioned that the preferred treatment for people with depression was psychosocial, and a small number 94(15.75%) of respondents mentioned that health facilities as the preferred treatment for people with depression.

More than one-third of respondents, 256(42.88%), identified depression as a mental illness while 215(36.01%) of them identified depression as the most serious mental illness (Table 2).

**Table 2:** Source of information about depression among respondents residing in AM-HDSS, Southern Ethiopia, 2019 (N = 617).

Variables	Category	Frequency	Percent
Ever heard about depression	Yes	597	96.80
	No	20	3.20
	Mass media	261	43.72
Main sources of information	Religious institutions	110	18.43
	From friends	489	81.91
	Family members	236	39.53
Depression is a mental health disorder	Yes	256	42.88
	No	341	57.12
Depression as the most serious mental disorders	Yes	215	36.01
	No	382	63.99
Known the cause of depression	Yes	393	65.83
	No	204	34.57
	Head injury	131	33.33
	Genetic	19	4.83
Perceived cause of depression (Multiple responses)	Physical illness	117	29.77
	Substance misuse	340	86.51
	Loss of loved one	240	51.91
	Conflict with family	184	46.82
	Punishment by God	50	12.72
	Evil spirit	122	31.20
	Poverty	121	31.11
	Aggression/destructiveness	45	7.54
Common manifestation of depression (Multiple responses)	Talkativeness	82	13.74
	Eccentric behavior	392	65.66
	Wandering	205	34.34
	Self-neglect	246	41.21
	Restlessness/anxiety	86	14.41
	Insomnia	178	29.82
	Loss of concision	83	13.90
Preferred place of treating mental illness	Family (Psychosocial)	430	72.03
	Spiritual/traditional	229	38.36
	Biological	300	50.25
	Health facilities	94	15.75
	Not treatable	4	0.67

### Community perception and attitude towards attitude towards people with depression:

The finding of this study shows that among the study participants, 39.9% (95% CI: 36.0–43.8%) had positive attitude towards people with depression and approximately half, 52.7 % (95% CI: 48.8 –56.6 %), of respondents have a good perception for people with depression (Table 3).

**Table 3:** Community Perception and Attitude towards people with depression among residents of Arba Minch Zuria District, AM-HDSS, Southern Ethiopia, 2019 (N = 617).

Variables	Category	Frequency	Percent
Community Perception towards	Good Perception*	325	52.7
people with depression	Poor Perception	292	47.3
Community attitude towards	Positive attitude*	246	39.9
people with depression	Negative attitude	371	60.1

* Good perception means who score ≤27 and Positive attitude means who score ≤39

### Community’s attitude towards people with depression:

The finding of this study found that more than half (63.3%) of study participants disagreed to a person who shows the signs of depression, should be hospitalized, and more than one third (38.4%) agreed that individuals with depression are simply weak-willed and unmotivated people. Also, 41.2% agreed that the main causes of depression are a lack of self-discipline and will-power. Nearly half (48.1%) agreed that if treated and medicated, people with depression can function fairly typically in society. More than half of the respondents (57.4%) disagreed that individuals with depression are victims of their disease and should be treated with sympathy while about 55.1% of the respondents disagreed that people with depression should have the same educational, occupational, and social opportunities as “normal” individuals. Nearly half (47.3%) of the participants agreed that anyone can suffer from depression (Table 4).

**Table 4:** Responses to the item the community’s attitude towards people with depression subscales among residents of Arba Minch Zuria District, AM-HDSS , Southern Ethiopia, 2019(N=617).

Items	Strongly Agree	Agree	Neutral	Disagree	Strongly Disagree
	N (%)	N (%)	N (%)	N (%)	N (%)
Do you agree as soon as a person shows signs of depression, he or she should be hospitalized?	14(2.3)	46(7.5)	86(13.9)	390(63.2)	81(13.1)
Do you agree one of the main causes of depression is a lack of self-discipline and will-power?	44(7.1)	254(41.2)	133(21.6)	161(26.1)	25(4.1)
Do you agree individuals with depression are simply weak-willed, unmotivated people?	49(7.9)	237(38.4)	115(18.6)	199(32.3)	17(2.8)
If treated and medicated, people with depression can function fairly typically in society?	112(18.2)	297(48.1)	66(10.7)	138(22.4)	4(0.6)
Depression reflects a response that is not amenable to change.	53(8.6)	239(38.7)	63(10.2)	241(39.1)	21(3.4)
Do you agree person with depression social problems are their fault because they isolate themselves from others?	44(7.1)	273(44.2)	103(16.7)	191(31)	6(1.0)
Do you agree most people with depression are poor?	25(4.1)	41(6.6)	60(9.7)	309(50.1)	182(29.5)
Do you agree depressed patients are more likely to have experienced deprivation in early life than other people?	48(7.8)	176(28.5)	85(13.8)	285(46.2)	23(3.7)
Do you agree individuals with depression do not need medication; they just need to change their thought processes and behaviors?	37(6.0)	232(37.6)	47(7.6)	222(36.0)	79(12.8)
Do you agree individuals with depression are victims of their disease and should be treated with sympathy?	26(4.2)	114(18.5)	101(16.4)	354(57.4)	22(3.6)
Do you agree people with depression have abnormal behavioral patterns?	81(13.1)	364(59.0)	103(16.7)	66(10.7)	3(0.5)
Do you agree people with depression should have the same educational, occupational, and social opportunities as “normal” individuals?	38(6.2)	130(21.1)	97(15.7)	340(55.1)	12(1.9)
Do you agree anyone can suffer from depression?	78(12.6)	114(18.5)	20(3.2)	292(47.3)	113(18.3)

### Factors Associated with Community Attitude towards people with depression:

The bi-variable logistic regression analysis showed that age of respondents, sex of respondents, education status of respondents, marital status of the respondents, perception towards people with depression, wealth index of respondents, having information about depression and family history of mental illness were found to be candidate variables for multivariable logistic regression analysis. After controlling the confounding effect, variables like educational status and marital status of respondents were significantly associated with community attitude towards people with depression. However, age, sex, perception towards people living with depression, wealth index, having information about depression, and family history of mental illness were not significantly associated with community attitude towards people with depression in a multivariable logistic regression model.

The finding of this study reveals that respondents who had no formal education were 1.82 times more likely to have a positive attitude towards people with depression compared to those who attended secondary and above *[AOR=1.82, 95% CI: 1.04,3.18]* education. The marital status of respondents was also significantly associated with attitudes towards people with depression. Those who were single during data collection time were 50% less likely to have a positive attitude towards people with depression compared to those who were married *[AOR=0.50, 95% CI: 0.28–0.89]* (Table 5).

**Table 5:** Factors associated with Community Attitude towards people with depression among residents of Arba Minch Zuria District, AM-HDSS, Southern Ethiopia, 2019.

Variables	Categories	Attitude towards depression		COR [95% CI]	AOR [95%CI]	p-value
		Positive	Negative			
Age in years	18–24	66(38.8)	104(61.2)	1.27(0.84,1.93)	0.94 (0.61, 1.44)	0.764
	25–44	93(36.9)	159(63.1)	1.38(0.94,2.02)	0.68 (0.42, 1.09)	0.105
	≥45	87(44.6)	108(55.4)	1.00	1.00	
Sex	Male	140(43.2)	184(56.8)	1.00	1.00	
	Female	106(36.2)	187(63.8)	1.34(0.97,1.86)	1.29 (0.91, 1.82)	0.156
Level of Education	No formal education	82(36.6)	142(63.4)	1.69(1.01,2.82)	***1.82(1.04, 3.18)***	0.040*
	Primary (1–8)	124(39.7)	188(60.3)	1.48(0.91,2.42)	1.44(0.86, 2.41)	0.17
	Secondary & Above	40(49.4)	41(50.6)	1.00	1.00	
Marital status	Married	215(38.5)	344(61.5)	1.00	1.00	
	Single	31(53.4)	27(46.6)	0.54(0.32,0.94)	**0.50(0.28, 0.89)**	0.020*
Family History of MI	Yes	19(42.2)	26(57.8)	1.00	1.00	
	No	227(39.7)	345(60.3)	1.00 0.90(0.49,1.67)	1.00 1.15 (0.61, 2.17)	0.656
Ever heard about MI	Yes	237(39.7)	360(60.3)	0.81(0.33,1.97)	1.42 (0.56, 3.61)	0.465
	No	9(45.0)	11(55.0)	1.00	1.00	
Perception to people with depression	Good	127(39.1)	198(60.9)	1.00	1.00	
	Poor	119(40.8)	173(59.2)	0.93(0.68,1.29)	1.08(0.77, 1.51)	0.663
Wealth index	Low	88(43.6)	114(56.4)	0.89(0.61,1.33)	0.81 (0.54, 1.22)	0.313
	Moderate	74(35.2)	136(64.8)	1.28(0.86,1.89)	1.19 (0.79, 1.78)	0.411
	High	84(41.0)	121(59.0)	1.00	1.00	

Note: MI (mental illness),

* p < 0.05; AOR adjusted odds ratio; COR crudes odds ratio; 1.00 reference category, CI confidence interval

## Discussion

The primary objective of this investigation was to determine the Arba Minch Zuria District residents’ perception and attitude towards depressed patients as well as to identify the factors associated with the status of study participant’s attitude for patients with depression. As a result, while nearly more than half of the residents had a good perception of people with depression, only two out of five individuals had a positive attitudes towards patients with depression. The study participants’ marital status and education level were significantly affected their attitude toward patients with depression. These findings imply that the significant proportion of the community members in the study area had poor perception and negative attitudes for patients with depression.

The proportion of the study participants with good perception for depressed patients in this study is relatively higher than those of residents in Hawassa city of Southern Ethiopia (9) but lower than the finding than the study conducted in Western Ethiopia among residents of Gimbi town (12). This difference might be due to the difference in the study settings of the study participants. This study was conducted in a rural community while the participants in other studies were towns and cities. Secondly, it might be due to the sample size difference.

Similarly, the percentage of individuals with positive attitude towards depressed patients in this study, 39.9% (95% CI: 36.00–43.8%), is higher than the finding from Hawassa city, Southern Ethiopia, 24.2% (9). This finding implies that there is a good community asset in the rural community for establishing and strengthening community-based rehabilitation for people living with a mental health problem. Besides, this finding may give hint for health professionals for better effectiveness of mental health programs through integrate patients into community-based services.

Furthermore, the individuals’ attitude for depressed patients is significantly associated with their marital status. Other studies similarly showed that married individuals have higher odds of having positive attitude for depressed patients than bachelor individuals (12,16). This might be because that being married increased social responsibility as well as an understanding of the situation through experience in the community.

The study participants’ level of education is also significantly associated with the status of attitude for patients with depression. According to the finding of this study, individuals with no formal education have a higher probability of being positive for depressed patients than individuals with formal education. However, this finding is inconsistent with the findings of the studies conducted in Riyadh, Saudi Arabia, Singapore, and Europe (16). This might be because that individuals in a developed country with an advanced educational level experienced more mental health-related situations from a long time and complicated working environment.

Moreover, substance misuse, loss of loved ones, and conflict within the family were perceived as the common causes of depression by the participants of this study. This finding concurred with the findings of the studies conducted in Hawassa city, Southern Ethiopia, Axum town and Jimma Town (9, 10, 17). It is a matter of fact that the identified situations exposed an individual to a stressful situation and thus increased the probability of developing depression in the long run.

Similar to the evidences from Agaro town, Gimbi town and Hawassa city of Ethiopia (9, 12, 18), psychosocial treatment was identified as the most preferred place for the treatment of depression. This finding may give hint on the need for awareness sessions for the community on possible treatment options for better effectiveness of modern antipsychotic treatment because the majority of people in our study area use traditional or psychosocial options as preferred methods.

In conclusion, this study found that more than half of the study participants had good perception for patients with depression but only two out of five study participants developed a positive attitude toward depressed patients. The study participants’ education and marital status significantly influenced the attitude toward depressed patients. Inappropriate use of substances, loss of intimate people as well as family conflict could expose individuals for depression. The most preferred treatment option for depression was psychosocial treatment.

Therefore, different stakeholders working on mental health programs can keep and potentially improve mental health services by improving community attitude and perception. Besides, working on streamlining and strengthening community awareness focusing on risk factors and preferred treatment options are also very important. Furthermore, the preventive mental health targeted interventions should consciously and closely focus on people who misuse substance, lost loved ones, and have conflict within a family to prevent the occurrence of depression. Moreover, further study should conduct by including other variables among rule residents.

As this study was exclusively conducted in the rural community, the findings cannot be generalized to all people living in Ethiopia. In addition, there may be social desirability bias on the information of attitude towards people with depression since we collected the data by using the interviewer-administered technique. The cause and effect relationships in this study could not be determined due to the use of a cross-sectional study design. Thus, we strongly recommend further study by employing a qualitative study design to understand well enough the community perception and attitude for patients with depression.
